# Physical Activity Knowledge, Attitude, and Behaviours Among Adolescents in the Kingdom of Saudi Arabia Prior to and during COVID-19 Restrictions

**DOI:** 10.1155/2022/1892017

**Published:** 2022-08-02

**Authors:** Naif Almutairi, Sharyn Burns, Linda Portsmouth

**Affiliations:** ^1^School of Population Health, Curtin University Bentley Campus, Perth, Western Australia, Australia; ^2^Department of Public Health, College of Health Sciences at Al-Leith, Umm Al-Qura University, Al-Leith, Saudi Arabia; ^3^Collaboration for Evidence Research and Impact in Public Health, School of Population Health, Curtin University, Perth, Western Australia, Australia

## Abstract

**Background:**

The prevalence of childhood and adolescent obesity has increased dramatically and poses a major public health threat globally. In the Kingdom of Saudi Arabia (KSA), the main cause of adolescent obesity is an increase in physical inactivity and unhealthy eating habits due to lifestyle changes. This study reports on factors associated with physical activity (PA) prior to and during the coronavirus disease of 2019 (COVID-19) among middle school students in Jeddah, KSA.

**Method:**

A cross-sectional online survey was conducted in Jeddah, KSA among 1500 middle school students aged 11 to 15 years. Sociodemographic characteristics; PA knowledge, attitude, and behaviours; and information about the impact of COVID-19 on PA were collected. Knowledge, attitude, and behaviours of PA before and during COVID-19 restrictions and between gender were compared. Regression analyses were conducted to assess the determinants of PA.

**Results:**

Female students were significantly more likely to report better knowledge of PA compared to males (*p* < 0.001). However, males were significantly more likely to participate in PA compared to females (*p* < 0.001). Approximately 60% of students reported their PA decreased during COVID-19 isolations. Most students did not participate in the recommended levels of daily PA during COVID-19 lockdowns and school closures. After adjusting for demographics, knowledge, and attitude, students who did not usually participate in school sports (*p*=0.017) and as members of clubs (*p*=0.002) were less likely to be active during COVID-19 lockdown.

**Conclusions:**

Efforts to enhance PA should be implemented through coordinated school and community-based programs to achieve the recommended PA among adolescents at home and in schools. Policy to ensure students receive PA opportunities at school is recommended.

## 1. Introduction

A preprint has previously been published.

Physical inactivity represents the fourth highest cause of premature and preventable death globally [1]. The risk of all causes of mortality increases by 20% to 30% for inactive compared to active people [2]. Inactive people are at increased risk of noncommunicable diseases [3] such as obesity [4], ischemic heart disease, diabetes [5], stroke [6], and numerous types of cancer [7].

Childhood and adolescent obesity is considered to be a major public health crisis globally. Globally, the prevalence of overweight among adolescents increased from 4% in 1975 to more than 18% in 2016 [3]. In 2019, it was estimated around 38 million children under the age of 5 were overweight or obese, and half of these children lived in Asia [8]. Overweight and obese children are at least two times more likely to be obese during adolescence and adulthood [9–11].

One cause of overweight and obesity among all age groups is the imbalance of calories consumed and expended [8]. This is primarily associated with decreased physical activity (PA) and increased intake of energy-dense foods [12]. A global WHO-led study of 1.6 million school-aged adolescents found more than 80% do not meet the minimum recommended level of PA and the percentage is higher among girls (85%) [13]. Physical inactivity is detrimental to adolescents' current and future health given the documented benefits of PA, which include improved cardiorespiratory circulation, muscular fitness, and metabolic health [14]. In addition, PA has been found to have a positive impact on cognitive performance, academic attainment, and mental health outcomes among adolescents [15, 16].

Prior to the industrialisation of the Kingdom of Saudi Arabia (KSA) (approx. 30 years ago), people led a simple and active life with the physical challenges of daily life demanding an active lifestyle [17]. However, the KSA has undergone significant lifestyle changes, including an increase in sedentary behaviours and unhealthy eating habits that have coincided with increases in the prevalence of overweight and obesity. These lifestyle changes have been associated with significant increases in noncommunicable diseases (NCDs) and their associated complications [18, 19]. National estimates of combined overweight and obesity prevalence among 13–18-year-old Saudi students were 36.6% and 38.4% for males and females, respectively [17]. Over 84% of Saudi males and 91.2% of female adolescents reported more than the recommended screen time (ST) (>2 hours) daily, and almost 50% of males and 75% of females did not meet daily PA guidelines of at least 60 minutes daily of moderate-to-vigorous intensity [20]. Sedentary behaviour among adolescents as a result of increased ST was also associated with a negative impact on eating behaviours irrespective of PA levels [21].

A previous study among Saudi adolescents in 2008 found good knowledge and a positive attitude towards PA to be associated with increased PA levels [22]. Adolescents' negative attitude towards adopting regular PA has been recognised as a barrier to PA behaviour later in life [23]. In addition to behaviour-related barriers, other identified barriers include: environmental (expense and inaccessibility of structures activities); school-based (limited PA time allocated, lack of variety of PA choices, and lack of teacher support); and policy (policy facilitating school-based PA) [24]. Cultural influences and beliefs in the Arabic-speaking region may also present a barrier to PA for females [25, 26].

The COVID-19 (SARS-CoV-2) pandemic and the associated shutdown of schools, organised activities, and public sports facilities in most countries have further impacted on PA behaviour [27]. This has resulted in significant changes in adolescents' daily habits and their opportunities to remain active [28]. Following the WHO declaration of COVID-19 as a pandemic on March 11, 2020, the Saudi Arabian government imposed a nationwide curfew to restrict movement for most of the daylight hours [29]. The Ministry of Health [30] outlined the following actions that were undertaken. In line with the WHO guidelines on dealing with the outbreak, Saudi Arabia closed multiple facilities including schools, gyms, and other sports clubs. Partial lockdowns were imposed between April 21 and May 11 and total lockdown from May 23 to May 27, 2020, which included the complete closure of all universities and educational institutions with a shift to online education. On May 28, the lockdown was partially lifted in all cities, the movement between regions was eased, and shopping malls were opened. The restrictions on domestic flights, restaurants and cafes, and parks were eased on May 31. The latest action was on June 21, when the partial lockdown was lifted in all regions. However, all levels of schools including higher education institutions continued online-based remote learning during 2020 [30].

PA participation for adolescents offers benefits for physical and psychological well-being, particularly during the pandemic era [31]. At this critical stage of their development, it is important for adolescents to achieve the recommended level of PA [32]. While WHO social distancing regulations were implemented to control the spread of COVID-19, it is essential to recognise that these restrictions have impacted adolescent PA and general well-being [33]. The aim of this study is to describe the self-reported PA knowledge, attitude, and behaviours of adolescent students in Jeddah, KSA and the associations between PA behaviours, and demographics, knowledge, and attitudes. The study compares behaviours before and during the COVID-19 pandemic. It also compares the knowledge, attitude, and behaviours of males and females concerning PA.

## 2. Methods

### 2.1. Procedures

This article reports findings from a broader study to explore knowledge, attitude, and behaviours around overweight and obesity among adolescent students in Jeddah, KSA [34]. The study was approved by the Curtin University of Human Research Ethics Committee (HR2020-0337) and permission was granted by the KSA Ministry of Education. Participant information sheets were provided to principals, parents, and students. Consent was obtained from the principals of each school involved, and individual consent and assent were obtained from parents and students, respectively. An online survey link using Qualtrics was sent to students via their school email accounts. Data collection took place from October 20, 2020 until November 11, 2020 as students were using online distance learning platforms at that time. No identifying data were collected from students.

To ensure the representativeness of the study population, the minimum sample size (*n*) required was estimated from the calculations below [35]:

(1) $$\begin{array}{c} n=\frac{Z1^{2}-\left(\alpha /2P\left(q\right)\right)}{d^{2}}, \end{array}$$

where Z1-*α*/2 is standard error when *α* = 0.05 (95% Confidence Interval) = 1.96; *Q* = 1−*P*; *P* is the prevalence of the attribute (23.1%); *d* is the acceptable difference using 5% (0.05); and *n* is the number of samples.

Based on this formula, after adjusting for a 10% nonresponse rate and considering the design effect (considered to be 2) [36],the minimum sample of intermediate school students aged 12–15 years was approximately 1200 (600 males and 600 females).

### 2.2. Setting

The study was conducted in six government schools (three boys' and three girls' Intermediate Schools) in Jeddah City, KSA among students from grades 7 to 9 (12 to 15 years). At the time of data collection, due to COVID-19 restrictions, schools were conducting online learning, hence the surveys were administered online. Qualtrics, a secure web-based survey software, was used to conduct the survey. After consent for school participation was obtained, the schools emailed 3483 students. Of those students, 815 (23.3%) participants (or their parents) did not provide assent or consent to participate resulting in an initial response rate of 76.6%. Of the surveys received, 1500 were complete, while 1168 (33.5%) were extensively incomplete (more than 50% incomplete items within the survey form). Incomplete questionnaires were excluded during the data cleaning process.

### 2.3. Instruments

Sociodemographic characteristics; PA knowledge, attitude, and behaviours; and information about the impact of COVID-19 on PA were collected. It was intended that body mass index (BMI) would be calculated by measuring students' height and weight [34]; however, this was not possible due to the requirement for online learning. During the COVID-19 pandemic, online surveys provide a useful platform to conduct research and ensure that the implementation of this project was feasible [37]. Where possible, the survey was developed using previously validated questions and scales and underwent content validity with education and health experts.

### 2.4. Measures

#### 2.4.1. Sociodemographic Characteristics

Sociodemographic characteristics included age, gender (male or female), and school level. Students were also asked about maternal and paternal education levels (primary, intermediate, high school, undergraduate, and postgraduate).

#### 2.4.2. Knowledge

The level of physical activity knowledge was assessed by five items which allowed “true,” “false,” and “I do not know” responses (score range 5 to 0; 5 highest). The knowledge score was then collapsed to high (≥4) or low (≤3). Similar to Azrin Shah et al. [38], these binary scores were calculated based on distributions relative to the median score of 4. The reliability of knowledge items was at the lower border of the acceptable level (Cronbach's alpha = 0.59).

#### 2.4.3. Attitude

Eight attitude items requiring a Likert scale response (5 items; strongly disagree to strongly agree) were included. Total scores were computed, and negative items were reverse coded (score range: 40-8; 40 represents the most positive attitude). The Cronbach's alpha test for the total PA-related attitude questions was 0.73. As for other studies, this scale was dichotomised to agree or disagree [39, 40]. The PA attitude item responses were explored using three levels of scales: the original five Likert scale, the dichotomisation of the scale, and the total continuous score derived from the Likert scale.

#### 2.4.4. Behaviour of Physical Activity before and during COVID-19

The survey was administered during a COVID-19 isolation period where students were online learning, hence students were asked to consider questions “as they relate to your daily life when you were at school before the COVID-19 outbreak.” A series of questions to determine regular involvement in physical activity at and outside school were included. Students were asked how they usually get to or from school. To measure physical activity opportunities at school, students were asked if their school has regular sports classes, if they participate in the classes, and if so, how often each week. Students reported if their school organised sports competitions in school and with other schools and if they participated in out of school recreational activities. Questions focused on regular involvement in; duration of participation; and type of sport. These questions were adapted from the Australian National Nutrition and Physical Activity Survey [41].

Physical activity during the COVID-19 restrictions was assessed by asking the number of days students participated in vigorous or moderate physical activity for at least 60 minutes during a week. This question has been validated previously [41]. Similar to other studies [42, 43], binary variables were created to represent less and more active students: <5 days per week (less active) and ≥5 days per week (more active). Other questions included comparative questions on PA, ST, social media use, and sleep changes during COVID-19 restrictions. Five response options ranged from “a lot less” to “a lot more.” An open-ended question regarding how the COVID-19 pandemic and related restrictions impacted participants' physical activity was included.

### 2.5. Statistical Analysis

Data were analysed using SPSS 25 (SPSS Inc., Chicago, IL, USA). All data were entered and checked through data exploration, out of range values were identified and corrected from the raw data. The outcome variables were examined using skewness and kurtosis scores to treat outliers, including distribution plots. Descriptive statistics were run for all independent and dependent variables and reported as mean, median, frequency, and percentage. Correlation testing was employed for continuous scores. Chi-squared tests were used to compare PA knowledge, attitude, and behaviours of physical activity before and during COVID-19 restrictions. Comparisons between male and female students for all variables were also done. Univariate and multivariate logistic regression analyses of students' moderate-to-vigorous PA less than five days during COVID-19 were conducted, and a crude odds ratio (COR) and adjusted OR (AOR) were determined. The significance level was set at *p*=0.05.

## 3. Results

### 3.1. Descriptive Results

Just over half of the participants were female (54%); the majority (75%) were aged 13 and 14 years; and 43%, 32%, and 25% were in grades 8, 7, and 9, respectively (Table 1). There was no statistical difference between genders for mother's education; however, there was a significant difference (*p*=0.034) between genders for father's education with female students' fathers being slightly more likely to have a tertiary degree compared to male students' fathers.

#### 3.1.1. Knowledge Items

A significantly higher proportion of female students knew that PA reduces stress compared to male participants (81.1% vs. 68.2, *p* < 0.001, respectively). Similarly, 70% of female and 63% of male students correctly identified the disadvantage of sitting for more than two hours (*p*=0.004). The median knowledge score was 4 (mean = 4.2) out of a possible score of 5 (interquartile range (IQR) = 1.1). Around half of the students (48.6%) answered all of the knowledge questions correctly with 25% correctly answering four out of five. Female students were significantly more likely than male students to correctly answer all questions (52.1% vs. 44.5%, *p* < 0.001, respectively) (Table 2).

#### 3.1.2. Attitude Items

Just under three-quarters of students agreed that people who regularly participate in physical activity live longer (65.6%) and that everyone should be physically active (83.5%). There were no significant differences between males and females for these questions. However, 38% of male students agreed that girls should not participate in physical activity compared to around 14% of girls (*p* < 0.001). Females (87%) were more likely than males (83%) to feel happy when participating in physical activity (*p*=0.021). More female students (81%) agreed (*p*=0.036) that regular physical activity increased blood circulation compared to males (77%). The total scores were calculated, and the minimum score recorded was 10 and a maximum of 40 with a median of 34 (IQR = 6) (Table 2).

### 3.2. Pre-COVID-19 Physical Activity Behaviour

The most common way to get to school was via private vehicle (65%), followed by bus (17%) and walking (15%) (Table 3). Among those who walked or rode a bike to and from school, 63% and 81%, respectively, were male students (*p* < 0.001). Only 65.5% of students indicated that their school held regular sports classes; and of these, 70.5% of students reported that they participated. About half of the students reported that their school organised sports competitions at school (female = 31%, male = 69%) and only 31% organised sports competitions with other schools (female = 30%, male = 69%, *p* < 0.001). Approximately 30% of students (female = 21%, male = 79%) participate in after-school sports (*p* < 0.001). Of these 10.6% of students (female = 35%, male = 65%) participate daily and 19.7% of students (female = 54%, male = 46%) participate most days. Only around 17% of students indicated they are members of organised sports clubs. Of these, 70% were males and 30% were females (*p* < 0.001).

**Figure 1:**
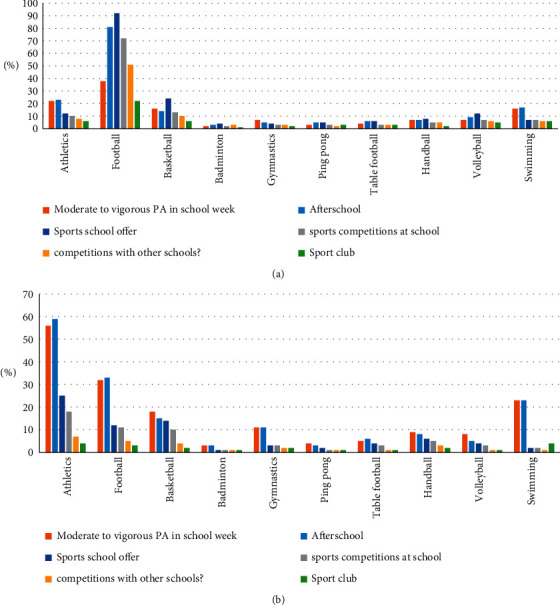
Sports played by students categorised by gender among 1500 adolescents in the KSA: (a) males and (b) females.

Football was the most popular sport played by male students (81%) as a moderate-to-vigorous PA during a school week, followed by athletics (23%) and swimming (17%) (Figure 1). Athletics (59%), football (33%), and swimming (23%) were the most popular moderate-to-vigorous PA during a school week among female students (Figure 1(b)). Similarly, football (54%), athletics (43%), and swimming (20%) were the three most common sports all students participated in after school (extracurricular activity). Football, athletics, swimming, and basketball were the most common sports that schools offer as part of intraschool and interschool competitions in both female and male schools. Overall, except for athletics, more males reported participating in each type of school and club activities compared to female students.

### 3.3. Perceived Physical Activity, ST, and Sleep Time during COVID-19 Restrictions

When asked about the impact of COVID-19 on their physical activity, 41% of students reported being a lot less active and 18% a little less active during isolation (Table 4). In contrast, students reported the time they spent watching television, movies, spending leisure time with computers and playing games increased a lot (26%) or a little (28%). About 22% and 26% of students spent a lot and a little more time on social media, respectively, during the COVID-19 isolation measures, while 28% reported time spent on social media did not change. Sleep patterns remained the same (38%), a little more (21%), and a lot more (15%) compared to prior to COVID-19 restrictions. While some students reported participating in more physical activity (19.2%), a further 21.6% indicated their level of activity did not change while 59% indicated they participated in less physical activity than before the isolation measures. Most responses to the open-ended question on the impact of COVID-19 isolation measures indicated a reduced level of PA due to the closure of gymnastic clubs, school sports clubs, or fear of becoming infected with the virus if they go to public places. Increased ST was also mentioned as a reason for lower PA levels.

Regular physical activity was low with 226 (20.3%) students participating in at least 60 minutes of moderate or vigorous PA for at least 5 days of the week. About 24% of students reported they never participated in any vigorous or moderate PA and 25% reported participating in PA on 1 day per week (Table 5).

### 3.4. Comparison of Least and More Active Students

Table 6 presents the association between participants' sociodemographic characteristics, knowledge, and attitude towards PA with the frequency of moderate-to-vigorous PA per week. Male participants (53.5%) were more likely to participate in regular moderate-to-vigorous PA compared to female students (46.5%; *p*=0.035). Among students who scored above the median knowledge score, 70.4% were more active compared to 77.5% of students reporting lower levels of PA (*p*=0.024). There was a significant difference (*p*=0.004) between students reporting high and lower levels of PA with regard to attitudes towards PA to manage the higher risk of some diseases (such as heart disease, diabetes, and high blood pressure) with students reporting lower PA levels more likely to agree with the statement (85.4% vs. 77.4%) (this is not included in Table 6). There were no other significant associations observed between students who participate in high levels of regular activity and those reporting lower levels with regard to other attitude items and other demographic characteristics.

A multivariable logistic regression model was employed, adjusted for all demographic, attitude score, knowledge score, and PA behaviour variables, to identify the predictors of less active students (Table 6). As the total knowledge score increased by one unit, the odds of students being less active increased (aOR = 1.14 (95% 1.04–1.40), *p*=0.014). Students who did not participate in afterschool sports were 1.6 times more likely to be less active (i.e., report less than 5 hours per week of moderate-to-vigorous exercise) compared to those who participated. Moreover, students who were not members of sports clubs were less active compared to students with sports club membership (aOR = 1.78 (95% CI: 1.23–2.57), *p*=0.002). Other demographic and attitude variables were not significantly identified as predictors of daily moderate-to-vigorous physical activity.

## 4. Discussion

The findings of this study describe physical activity knowledge, attitude, and behaviours of adolescents aged 11–15 years from Jeddah, KSA prior to and during COVID-19 restrictions. Sociodemographic characteristics, PA knowledge, attitude, and behaviours were stratified by gender. Male and female students included in this study were comparative. Findings revealed students to have good knowledge about the health benefits of physical activity with female students reporting better knowledge overall than males. Our study results are similar to previous research conducted in Iran where female middle school students reported significantly higher mean scores of PA knowledge than their male colleagues [44].

Globally, the majority of adolescents do not fulfil the WHO recommended amount of daily PA of 60 minutes or more of moderate-to-vigorous physical activity daily [14]. This study also found most students did not participate in the recommended levels of daily PA, with females reporting significantly lower rates than males. A previous study in Saudi Arabia found only 44% of males and 20% of female adolescents to be adequately active [45]. While this study collected data during COVID-19 restrictions, students were also asked to reflect on a usual activity during a usual face-to-face school week. Females in this study were less likely to be involved in PA even though female schools offer a range of PA opportunities and sports competitions. However, when asked if girls should participate in PA, the majority of females (86.1%) agreed compared to only 61.8% of boys agreeing girls should participate. Others have found adolescent females in the KSA have limited opportunities for involvement in sports and recreational activities due to cultural and gender norms. The cultural context in the KSA may discourage females from becoming physically active [26, 46]. The findings of this study demonstrate while females agree participation in PA is important, most are not participating at desired levels. There is a need for multicomponent, targeted interventions to enhance the acceptability of and participation in PA among adolescent girls in the KSA. A study of Czech and Polish adolescents recommended interventions for girls should focus on enhancing enjoyment and competence. In addition, appearance motives can also be employed to motivate girls [47].

In addition, students in this study reported they usually travelled to and from school via cars and/or bus, reducing incidental PA opportunities. Although overall levels were low, more males walked or rode their bike to and from school compared to female students. Saudi Arabia's hot dry year-round climate, with temperatures often rising above 50°C (122°F) during the hot season, likely discourages students from walking, cycling, or using scooters in an effort to avoid exposure to sunlight and heat stroke [48]. Despite the advantages of incidental exercise, an Iranian study found contribution to moderate-to-vigorous PA activity was limited; however, the authors recommended encouraging students to walk to school and to complement this activity with other structured PA [49].

Approximately 60% of adolescents in this study reported they did “less” or a “lot less” PA level than usual during the COVID-19 restrictions. Similarly, other studies have also found adolescent PA levels have significantly decreased while daily ST has increased during COVID-19 restrictions [50, 51]. Adolescents and children have been found to be more likely to adhere to healthy behaviours on structured days, for example school days, compared to unstructured days such as weekends or holidays [52]. COVID-19 restrictions have caused multiple organisations, clubs, gyms, and schools to close or reduce activities, resulting in adolescents missing opportunities for regular PA [53]. Restrictive measures have provided the opportunity to explore other ways to engage adolescents including web home-based PA, online physical education, and active video games [47, 54].

Sociodemographic characteristics such as age and gender play an important role in shaping attitudes and behaviours towards PA and ST [45, 55] as well as their propensity to show changes during this pandemic [56]. In this study, females were more likely to report lower levels of PA prior to and during COVID-19 compared to males. Students who were not participating in after school sports and those who were not members of sports clubs before COVID-19 were more likely to be less active during COVID-19. Regardless of the students' knowledge, attitude, and PA behaviours prior to COVID-19 restrictions, social isolation and imposed rules to stay at home for a prolonged period have negatively impacted PA levels and sedentariness. PA has numerous physical fitness benefits, in addition to increasing positive mood; promoting self-esteem; and reducing stress, anxiety, and depression [56, 57], hence it is important opportunities to maintain activity are sustained during periods of restrictions. Restrictions may also provide an opportunity to engage less active adolescents through the use of online activity programs [58].

A strength of this study was the 77% response rate which is considered an excellent rate for an online survey [59]. Despite its novel contributions to adolescent PA knowledge, attitude, and behaviours during and pre-COVID-19-related restriction in KSA, this study has some limitations. First, in the cross-sectional design, the temporal link between outcome and exposure cannot be established or imply causation as both are examined simultaneously. The study also used self-report PA behaviours and asked students their perception of how COVID-19 impacted their PA level, which may be subject to social desirability or recall bias. Third, it was originally intended to measure BMI [34], which would have enabled analysis between activity levels and body size; however, this was not possible due to online schooling during the data collection period.

## 5. Conclusion

This study found while most students have good knowledge of the importance of PA, and their attitudes towards PA were positive, the majority of students did not meet recommended levels of PA. When PA opportunities were considered prior to COVID-19 restrictions, participation levels remained low, especially for females. Knowledge of the health benefits of PA is desirable and should be maintained, developed, and improved by well-organised school health education and health promotion programs. More efforts to enhance attitudes towards PA and to provide opportunities for regular structured PA, especially for females, should be encouraged [60]. Coordinated school and community-based programs are recommended. School-based and governmental policy to provide guidelines for regular PA opportunities would enhance PA opportunities for students. Further, given the likelihood of ongoing restrictions associated with COVID-19 schools, community groups and families should encourage adolescents to remain physically active through the use of virtual community classes, creating challenges with family members, and introducing PA during online school-based classes.

## Figures and Tables

**Table 1:** Sociodemographic characteristics among 1500 adolescents in the KSA by gender.

Variables	Levels	Frequency	Male *n* = 688 (45.6%)	Female *n* = 822 (54.4%)	*p*-value
Demographic info		*n* (%)	*n* (%)	*n* (%)	
Age	11	35 (2.3)	16 (2.3)	19 (2.3)	<0.001^*∗∗∗*^
	12	250 (16.6)	88 (12.8)	162 (19.7)	
	13	466 (30.9)	222 (32.3)	244 (29.7)	
	14	658 (43.6)	357 (51.9)	301 (36.6)	
	15	101 (6.7)	5 (0.7)	96 (11.7)	
Grade	7	485 (32.1)	173 (25.1)	312 (38.0)	<0.001^*∗∗∗*^
	8	645 (42.7)	363 (52.8)	282 (34.3)	
	9	380 (25.2)	152 (22.1)	228 (27.7)	
Maternal level of education	<Primary	276 (18.3)	133 (19.3)	143 (17.4)	0.470
	Intermediate	193 (12.8)	92 (13.4)	101 (12.3)	
	High school	447 (29.6)	195 (28.3)	252 (30.7)	
	Undergraduate	513 (34.0)	226 (32.8)	287 (34.9)	
	Postgraduate	81 (5.4)	42 (6.1)	39 (4.70)	
Paternal level of education	<Primary	513 (34.0)	102 (14.8)	115 (14.0)	0.034^*∗*^
	Intermediate	81 (5.4)	80 (11.6)	83 (10.1)	
	High school	444 (29.4)	210 (30.5)	234 (28.5)	
	Undergraduate	515 (34.1)	207 (30.1)	308 (37.5)	
	Postgraduate	171 (11.3)	89 (12.9)	82 (10.0)	

*p*-value for chi-squared test; ^*∗*^*p* < 0.05; ^*∗∗*^*p* < 0.01; ^*∗∗∗*^*p* < 0.001.

**Table 2:** Knowledge and attitude towards physical activity among 1500 adolescents in the KSA by gender.

Variables	Levels	Frequency	Male *n* = 688 (45.6%)	Female *n* = 822 (54.4%)	*p*-value
Knowledge items		*n* (%)	*n* (%)	*n* (%)	
Regular physical activity can help build strong bones and muscles	False/do not know	97 (6.4)	44 (6.4)	53 (6.4)	0.967
	True	1413 (93.6)	644 (93.6)	769 (93.6)	
Regular physical activity can prevent obesity	False/do not know	121 (8.0)	59 (8.6)	62 (7.5)	0.462
	True	1389 (92.0)	629 (91.4)	760 (92.5)	
Regular physical activity leads to a lower risk of diseases	False/do not know	276 (18.3)	134 (19.50)	142 (17.3	0.270
	True	1234 (81.7)	554 (80.5)	680 (82.7)	
Physical activity can reduce stress	False/do not know	374 (24.8))	219 (31.8)	155 (18.9)	<0.001^*∗∗∗*^
	True	1136 (75.2)	469 (68.2)	667 (81.1)	
Sitting for more than 2 hours is bad for bones and muscles	False/do not know	496 (32.8)	252 (36.6)	244 (29.7)	0.004^*∗∗*^
	True	1014 (67.2)	436 (63.4)	578 (70.3)	
Knowledge scores	≤2	141 (9.3)	82 (11.9)	59 (7.2)	<0.001^*∗∗∗*^
	3	244 (16.2)	131 (19.0)	113 (13.7)	
	4	391 (25.9)	169 (24.6)	222 (27.0)	
	5	734 (48.6)	306 (44.5)	428 (52.1)	

Attitude items					
People who regularly participate in physical activity live longer than those who do not	Agree	990 (65.6)	467 (67.9)	523 (63.6)	0.083
	Disagree	520 (34.4	221 (32.1)	299 (36.4)	
Everyone should participate in physical activity	Agree	1261 (83.5)	583 (84.7)	678 (82.5)	0.239
	Disagree	249 (16.5)	105 (15.3)	144 (17.5)	
I dislike participating in physical activity	Agree	457 (30.3)	206 (29.9)	251 (30.5)	0.803
	Disagree	1053 (69.7)	482 (70.1)	571 (69.5)	
Girls should not participate in physical activity	Agree	377 (25.0)	263 (38.2)	114 (13.9)	<0.001^*∗∗∗*^
	Disagree	1133 (75.0)	425 (61.8)	708 (86.1)	
Regular participation in physical activity makes me healthy	Agree	1425 (94.4)	645 (93.8)	780 (94.9)	0.338
	Disagree	85 (5.6)	43 (6.3)	42 (5.1)	
Physical activity can be used to manage the higher risk of some diseases such as heart disease, diabetes, and high blood pressure	Agree	1256 (83.2)	562 (81.7)	694 (84.4)	0.156
	Disagree	254 (16.8)	126 (18.3)	128 (15.6)	
When I participate in physical activity, I feel happy	Agree	1290 (85.4)	572 (83.1)	718 (87.3)	0.021^*∗*^
	Disagree	220 (14.6)	116 (16.9	104 (12.7)	
Regular physical activity increases blood circulation	Agree	1195 (79.1)	528 (76.7)	667 (81.1)	0.036^*∗*^
	Disagree	315 (20.9)	160 (23.3)	155 (18.9)	

*p*-value for chi-squared test; ^*∗*^*p* < 0.05; ^*∗∗*^*p* < 0.01; ^*∗∗∗*^*p* < 0.001.

**Table 3:** Physical activity behaviour among students (pre-COVID-19 restrictions) among 1500 adolescents in the KSA.

Items		Total *n* (%)	Male *n* (%)	Female *n* (%)	*p*-value
How do you usually get to or from school?					
Walk	Yes	238 (15.8)	150 (63.0)	88 (37.0)	<0.001^*∗∗∗*^
Ride skateboard/scooter/rollerblades	Yes	17 (1.1)	9 (52.9)	8 (47.1)	0.539
Private vehicle (incl. taxi)	Yes	988 (65.4)	400 (40.5)	588 (59.5)	<0.001^*∗∗∗*^
Ride bike	Yes	36 (2.4)	29 (80.6)	7 (19.4)	<0.001^*∗∗∗*^
Bus	Yes	259 (17.2)	92 (35.5)	167 (64.5)	<0.001^*∗∗∗*^
Does your school have regular sports classes?	Yes	989 (65.5)	669 (67.6)	320 (32.4)	<0.001^*∗∗∗*^
	No	521 (34.5)	19 (3.6)	502 (96.4)	
If yes, do you participate?	Yes	697 (70.5)	481 (69.0)	216 (31.0)	0.014^*∗*^
	No	53 (5.4)	42 (79.2)	11 (20.8)	
	Sometimes	239 (24.2))	146 (61.1)	93 (38.9)	
How often each week?	None	574 (38.0	61 (10.6)	513 (89.4)	<0.001^*∗∗∗*^
	<1 hour	282 (18.7)	173 (61.3)	109 (38.7)	
	1 hour	343 (22.7)	203 (59.2)	140 (40.8)	
	1-2 hours	159 (10.5)	119 (74.8)	40 (25.2)	
	2-3 hours	65 (4.3)	54 (83.1)	11 (16.9)	
	>3 hours	87 (5.8)	78 (89.7)	9 (10.3)	
Does your school organise sports competitions at school?	Yes	756 (50.1)	522 (69.0)	234 (31.0)	<0.001^*∗∗∗*^
	No	754 (49.9)	166 (22.0)	588 (78.0)	
Does your school organise sports competitions with other school?	Yes	470 (31.1)	373 (79.4)	97 (20.6)	<0.001^*∗∗∗*^
	No	1040 (68.9)	315 (30.3)	725 (69.7)	
Do you participate in after school sports	Yes	456 (30.2)	297 (65.1)	159 (34.9)	<0.001^*∗∗∗*^
	No	476 (31.5)	188 (39.5)	288 (60.5)	
	Sometimes	578 (38.3)	203 (35.1)	375 (64.9)	
How often?	Daily	160 (10.6)	104 (65.0)	56 (35.0)	<0.001^*∗∗∗*^
	Most days	298 (19.7)	138 (46.3)	160 (53.7)	
	Some days	576 (38.1)	258 (44.8)	318 (55.2)	
	None	476 (31.5)	188 (39.5)	288 (60.5)	
For how long?	<hour	256 (17.0)	91 (35.5)	165 (64.5)	<0.001^*∗∗∗*^
	1 hour	383 (25.4)	171 (44.6)	212 (55.4)	
	1-2 hours	279 (18.5)	162 (58.1)	117 (41.9)	
	2-3 hours	68 (4.5)	47 (69.1)	21 (30.9)	
	>3 hours	48 (3.2)	29 (60.4)	19 (39.6)	
	None	476 (31.5)	188 (39.5)	288 (60.5)	
Member of a sports club	Yes	254 (16.8)	178 (70.1)	76 (29.9)	<0.001^*∗∗∗*^
	No	1256 (83.2)	510 (40.6)	746 (59.4)	

*p*-value for chi-squared test; ^*∗*^*p* < 0.05; ^*∗∗*^*p* < 0.01; ^*∗∗∗*^*p* < 0.001. ^*∗*^Total *n* varies between questions.

**Table 4:** Physical activity, ST, and sleep time during COVID-19 (row percentage) activity among 1500 adolescents in the KSA.

COVID-19 items	A lot less	A little less	About the same	A little more	A lot more
Have you participated in physical activities or sports?					
Male	326 (48.6)	114 (17.0)	137 (20.4)	53 (7.9)	41 (6.1)
Female	278 (34.7)	153 (19.1)	181 (22.6)	111 (13.9)	78 (9.7)
Have you watched TV and movies, used the computer for leisure, or played video games?					
Male	106 (15.8)	73 (10.9)	139 (20.7)	178 (26.5)	175 (26.1)
Female	110 (13.7)	71 (8.9)	179 (22.3)	234 (29.2)	207 (25.8)
Have you used social media?					
Male	108 (16.1)	78 (11.6)	193 (28.8)	164 (24.4)	128 (19.1)
Female	85 (10.6)	72 (9.0)	227 (28.3)	216 (27.0)	201 (25.1)
How was your sleep?					
Male	97 (14.5)	94 (14.0)	263 (39.2)	141 (21.0))	76 (11.3)
Female	84 (10.5)	111 (13.9)	291 (36.3)	174 (21.7	141 (17.6)

**Table 5:** The distribution of moderate-to-vigorous physical activity during COVID-19 restriction among 1500 adolescents in the KSA.

	Level	Frequency *n* (%)	Male *n* (%)	Female *n* (%)	Chi-square *p*-value
Vigorous or moderate physical activity for at least 60 minutes/week	None	352 (24.0)	140 (39.8)	212 (60.2)	0.107
	1	363 (24.8)	171 (47.1)	192 (52.9)	
	2	259 (17.7)	117 (45.2)	142 (54.8)	
	3	182 (12.4)	81 (44.5)	101 (55.5)	
	4	82 (5.6)	36 (43.9)	46 (56.1)	
Missing 46 (3%)	5	75 (5.1)	38 (50.7)	37 (49.3)	
	6	28 (1.9)	14 (50.0)	14 (50.0)	
	7	123 (8.4)	69 (56.1)	54 (43.9)	
	≥5 days/week	226 (20.3)	121 (53.5	105 (46.5	
	<5 days/week	886 (79.7)	405 (45.7	481 (54.3	

**Table 6:** Comparing least active students versus more active students among 1500 adolescents in the KSA.

Variables	Moderate-to-vigorous exercise (≥60 m·min.)		Chi-square (*p*-value)	Adjusted odds ratio (aOR) (Confidential interval (CI))	*p*-value
	≥5 days/week *N* = 226 (20.3%)	<5 days/week *N* = 886 (79.7%)			
Age					
11–12	34 (15.0)	179 (20.2)	0.212	1.69 (0.97–2.94)	0.065
13	70 (31.0)	260 (29.3)		1.09 (0.73–1.62)	0.688
14-15	122 (54.0)	447 (50.5)		1	
Gender					
Male	121 (53.5)	405 (45.7)	0.035	1	
Female	105 (46.5)	481 (54.3)		1.00 (0.68–1.47)	0.987
Grade					
7	74 (32.7)	289 (32.6)	0.918	0.77 (0.45–1.30)	0.324
8	94 (41.6)	380 (42.9)		1.13 (0.75–1.70)	0.562
9	58 (25.7)	217 (24.5)			
Maternal level of education					
<Primary	45 (19.9)	165 (18.6)	0.589	1.42 (0.69–2.95)	0.344
Intermediate	28 (12.4)	116 (13.1)		1.56 (0.74–3.29)	0.243
High school	62 (27.4)	251 (28.3)		1.39 (0.72–2.70)	0.327
Undergraduate	72 (31.9)	304 (34.3)		1.45 (0.76–2.76)	0.258
Postgraduate	19 (8.4)	50 (5.6)		1	
Paternal level of education					
<Primary	36 (15.9)	121 (13.7)	0.637	0.93 (0.49–1.78)	0.833
Intermediate	25 (11.1)	89 (10.0)		0.94 (0.49–1.81)	0.850
High school	62 (27.4)	255 (28.8)		1.08 (0.63–1.85)	0.784
Undergraduate	71 (31.4)	315 (35.6)		1.15 (0.69–1.92)	0.596
Postgraduate	32 (14.2)	106 (12.0)		1	
Knowledge score					
Above median	159 (70.4)	687 (77.5)	0.014	1.21 (1.04–1.40)	0.020^*∗*^
Below median	67 (29.6)	199 (22.5)			
Participation in school sport					
All the time	131 (58.0)	430 (48.5)	0.040	1	
Sometimes	29 (12.8)	134 (15.1)		1.11 (0.73–1.68)	0.626
No	66 (29.2)	322 (36.3)		1.15 (0.72–1.86)	0.559
Participation in after school sports					
All the time	103 (45.6)	270 (30.5)	<0.001	1	
Sometimes	75 (33.2)	369 (41.6)		1.60 (1.05–2.45)	0.029^*∗*^
No	48 (21.2)	247 (27.9)		1.59 (1.09–2.31)	0.017^*∗*^
Member of sports club					
Yes	70 (31.0)	144 (16.3)	<0.001	1	
No	156 (69.0)	742 (83.7)		1.78 (1.23–2.57)	0.002^*∗*^

^#^ Scores of knowledge and attitude (continuous variable) were used to fit in the model; ^*∗*^*p* < 0.05; ^*∗∗*^*p* < 0.01; ^*∗∗∗*^*p* < 0.001. ^*∗*^Total number of students may vary.

## Data Availability

The data that support the findings of this study are available on request from the corresponding author.
